# Improving osseointegration of Co-Cr by nanostructured titanium coatings

**DOI:** 10.1186/2193-1801-3-197

**Published:** 2014-04-21

**Authors:** Vuong-Hung Pham

**Affiliations:** Advanced Institute for Science and Technology (AIST), Hanoi University of Science and Technology (HUST), No 01, Dai Co Viet road, Hanoi, Vietnam

**Keywords:** Biomaterials, Sputtering, Coatings, Alloys, Thin films, Metal

## Abstract

This study reports the deposition of nanostructured Ti films on Co-Cr substrates to improve their surface characteristics and biocompatibility. The microstructure of the Ti films was controlled by application of negative substrate bias voltages. The surface roughness of Co-Cr implants was increased significantly after Ti coatings. The nanostructured Ti films are found to improve osteointergration of Co-Cr implants as indicated by enhancing cellular attachment, proliferation and differentiation, which was attributed mainly to the application of a biocompatible Ti coating, possessed a higher surface area for cell attachments and growth.

## Introduction

Co-Cr is the most extensively studied metallic biomedical implant due to its outstanding properties such as high strength, high corrosion resistance, flexibility and biocompatibility (Ohmori et al. 2006; Reclare et al. 2005). However, lack of osseointegration is limited its application (Okazaki and Gothoh 2005; Granchi et al. 1999; Ingham and Fisher 2005). Previous studies have shown that surface modification of Co-Cr by coating their surface with a bioinert material such as diamond-like carbon (DLC) (Choubey et al. 2004), titanium oxide (TiO_2_) (Han et al. 2009; Dicu et al. 2008) and titanium nitride (TiN) (Pham et al. 2011a, b) would offer improved the osseointegration, supporting bone growth on the Co-Cr implants. Nevertheless, there is a concern about instability of the coating-substrate interface because there are a lot of differences in physical and chemical properties between the coating and the substrates.

Ti and its alloys are some of the most biocompatible metals and they have been proven to be the effective materials for improving the osseointegration properties *in vitro* (Jayaraman et al. 2004; Citeau et al. 2005) and even *in vivo* (Li et al. 2010; Matsuno et al. 2001). In recent years, researchers have been shown that nanostructured Ti coating can enhance the biocompatibility of biomedical metals significantly (Vetrone et al. 2009; Khang et al. 2008) because of its high surface area (Liu et al. 2007), which provided higher binding sites and interlocking for osteoblast growth (Khang et al. 2012; Venkatsurya et al. 2012; Kim et al. 2012). More recent studies reported that nanoscale surface roughness, which directly correspond to the size of protein and cell membrane receptor, could also be sensitive to osteoblast proliferation and differentiation *in vitro* (Lipski et al. 2008; Webster et al. 2000), bioactive *in vivo* (Xue et al. 2005), protein adhesion (Dolatshahi-Pirouz et al. 2010) and gene expression (Mendonca et al. 2009). Nevertheless, there are no reports on the effect of nanostructured Ti coating on Co-Cr alloy for potential applications as the orthopaedic and dental implants.

Therefore, in this study, a Ti film was deposited on a Co-Cr substrate by DC sputtering. The microstructure and the surface roughness of the Ti films deposited Co-Cr substrates were evaluated (FE-SEM) and atomic force microscopy (AFM) testing, respectively. Pre-osteoblast (MC3T3-E1) were used for an osseointegration evaluation in terms of cell attachment, proliferation and differentiation and compared with those of the uncoated Co-Cr.

## Materials and methods

Ti films were deposited onto Co-Cr substrates by DC sputtering (Ultech, Daegu, Korea). Prior to deposition, the Co-Cr substrates (Bukang Coalloy, Korea) with dimensions of 10 mm × 10 mm × 1 mm or 20 mm × 20 mm × 1 mm were ground with a 2000-grit SiC abrasive paper and cleaned ultrasonically. The deposition chamber was pumped to 5×10^−4^ Pa using rotary and diffusion pumps. The substrate was then subjected to ion bombardment in an argon flow discharge under a negative bias voltage of 600 V for 30 min to remove any residual surface contamination. Subsequently, the Ti films were deposited by DC sputtering of a Ti target (diameter 75 mm, thickness 5 mm, purity 99.99%, Kahee Metal, Korea) at a deposited power of 60 W in high purity argon (99.998% pure). The sputtering process of Ti film was carried out by varying the application of substrate bias voltages (V_b_) up to 100 V to the Co-Cr substrate to control the structure of Ti films. The deposition of Ti films was carried out without the application of a negative substrate bias to the Co-Cr substrate. For the Ti films studied herein, the working pressure of 0.6 Pa were employed during reactive sputtering, while the substrate temperature of 100°C was maintained using a halogen heater with a programmable temperature controller.

The microstructure and surface morphology of Ti films deposited on Co-Cr substrates were studied by field emission scanning electron microscopy (SUPRA 55 VP, CARL ZEISS, Germany) operated at 2 kV. In addition, the surface morphology and average surface roughness (RMS) of the samples were measured by atomic force microscopy (Nanostation II, Germany) in tapping mode with a 5-μm scan sizes for both x and y axis.

Pre-osteoblasts MC3T3-E1 (ATCC, CRL-2593) were used to examine the interaction between the cell and specimens (uncoated Co-Cr substrate and Ti-deposited Co-Cr). The cells were maintained in α-MEM containing 10% fetal bovine serum (FBS) and 1% antibiotic at 37°C in humidified air and 5% CO_2_. The cell cytoskeleton organization was visualized by confocal laser scanning microscopy (CLMS, Zeiss-LSM510, Carl Zeiss Inc., NY, USA). After culturing for 24 h and 72 h, the cells on the tested sample were fixed in 4% paraformaldehyde in PBS for 10 min, washed in PBS, permeabilized with 0.1% Triton X-100 in PBS in 7 min, washed in PBS and stained with fluorescent anti-tubulin for 30 min. The cell nuclei were counterstained with DAPI for 5 min. The stained samples were placed on a cover slide, and the cell morphology was observed.

The rate of proliferation was measured after culturing for up to 10 days using 3-(4,5-dimethylthiazol-2-yl)-5-(3-carboxy-methoxyphenyl)-2-(−4-sulfophenyl)-2H-tetrazolium (MTS, Promega, Madison, WI, USA) for mitochondrial reduction. The cells (2 × 10^4^ cell/mL) were seeded on the specimens (uncoated Co-Cr substrate and Ti-deposited Co-Cr) and cultured for 10 days. They were then washed with PBS and placed in a culture medium containing the MTS solution and returned to the incubator at 37°C for 3 h. This assay is based on the ability of metabolically active cells to reduce a tetrazolium-based compound, MTS, to a purple formazan product. The quantity of formazan product, which is measured by the absorbance at 490 nm using a micro-reader (Biorad, Model 550, USA), is directly proportional to the number of living cells in the culture.

The extent of cell differentiation was assessed by measuring the alkaline phosphatase (ALP) activity of the cells cultured on the specimens (uncoated Co-Cr substrate and Ti-deposited Co-Cr). The cells (1 × 10^4^ cell/mL) were seeded on the specimens and cultured for 21 days. They were then washed with PBS and detached using trypsin-ethylene diamine tetraacetic acid. The amount of protein in the cell lysates was quantified using a protein assay kit (biorad, Hercules, CA, USA) and the ALP activity was assayed calorimetrically using p-nitrophenyl phosphate (pNPP, Sigma-Aldrich, UK). This colorimetric assay is based on the conversion of pNPP to *p*-nitrophenol (pNP) in the presence of ALP, where the rate of pNP production is proportional to the ALP activity. The absorbance of the reaction product, p-nitrophenol, was measured at 405 nm using a microplate reader.

The data is presented as the mean ± standard deviation. Statistical analysis was performed using a t-test. A p value < 0.05 was considered significant.

## Results and discussion

**Figure 1:**
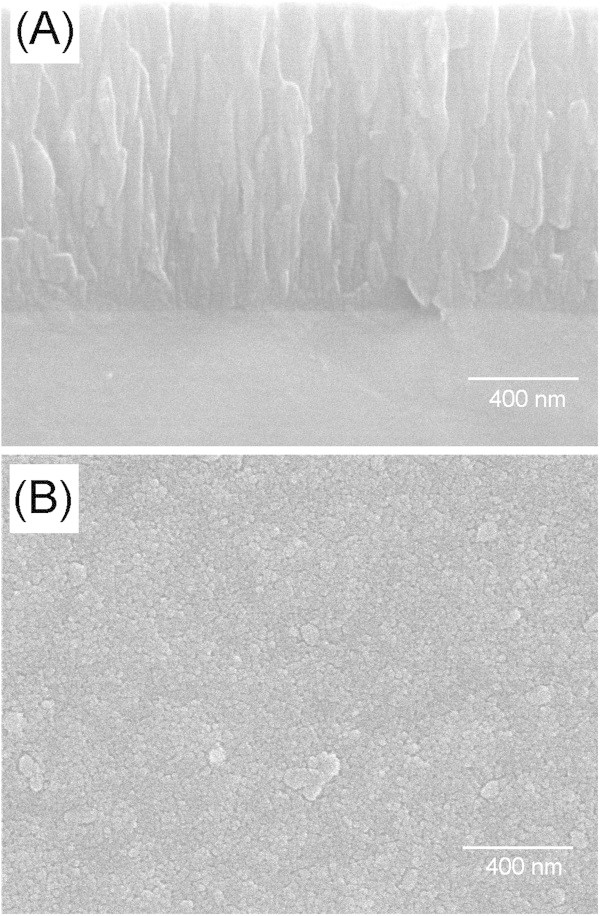
**SEM images showing (A) cross-section and (B) surface morphology of the Ti-deposited Co-Cr.**

The microstructure of the Ti film on the Co-Cr substrate was examined by SEM, as shown in Figure 1(A) and (B). The Ti film showed a dense and uniform layer with a thickness of ~ 2 μm (Figure 1(A)). In addition, there was good adhesion between the Ti film and Co-Cr alloy substrate, which was attributed to the use of a thin refractory Ti metal as coating materials. The surface of the Ti film was represented by fine grains without noticeable voids or cracks (Figure 1(B)).

**Figure 2:**
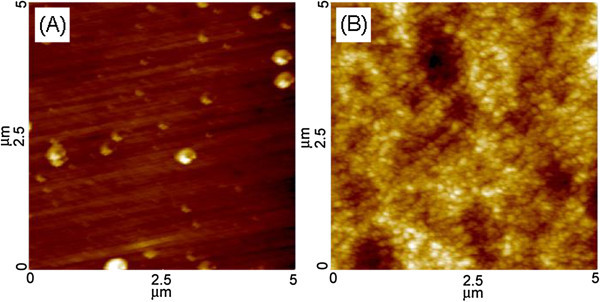
**AFM images of (A) as-polished Co-Cr and (B) Ti-deposited Co-Cr.**

The surface morphology of the Ti film was examined more closely by AFM. Figure 2(A) and (B) show typical AFM images of the surface morphology of the Co-Cr substrate and Ti-deposited Co-Cr, respectively. The surface roughness of the Co-Cr substrate was 1.97 nm (Figure 2(A)), whereas the value on the Ti-deposited Co-Cr was 5.76 nm (Figure 2(B)). This increase in surface roughness after Ti deposition would be expected to enhance the biocompatibility of the Co-Cr implants.

**Figure 3:**
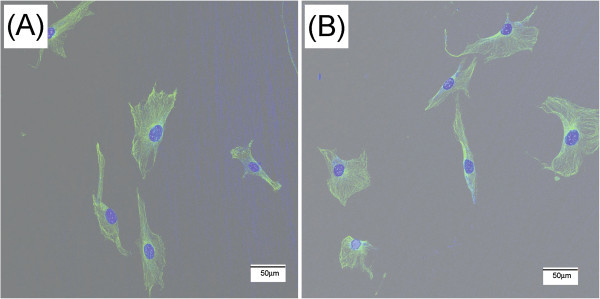
**Microtubule organization of cell on the tested specimens after 24 h of culturing. (A)** uncoated Co-Cr and **(B)** Ti-deposited Co-Cr.

The biological properties of the Ti-deposited Co-Cr were evaluated and compared with those of the uncoated Co-Cr substrate. Figure 3(A)-(B) show the representative CLMS micrographs of microtubules organization on the Co-Cr substrates and the Ti-deposited Co-Cr after 24 h of seeding. After 24 h seeding, cells began to spread on the tested surfaces. Osteoblasts cells cultured on bare Co-Cr and Ti-deposited Co-Cr displayed well organization of microtubules, but with different patterns and levels. Osteoblast on Ti-deposited Co-Cr showed bigger cell shape and well defined patterns of tubulin represented by more microtubles in the cells compared to that bare Co-Cr.

**Figure 4:**
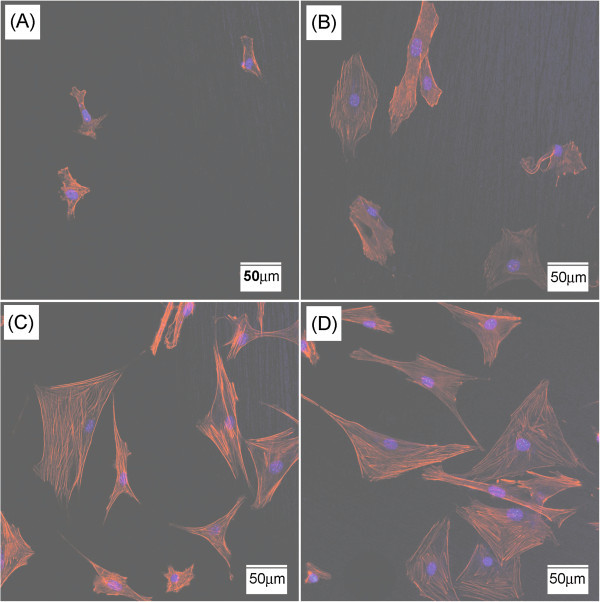
**Actin stress fiber organization of cell on the tested specimens after 24 h and 72 h of culturing. (A,C)** uncoated Co-Cr, **(B,D)** Ti-deposited Co-Cr. **(A-B)** 24 h, **(C-D)** 72 h of culturing.

We further analyzed the cytoskeleton of the osteoblasts by observing actin stress fiber of the cells. Examination of actin focused on the analyzing the cells cultured on the films to formed stress fiber. Figure 4(A)-(D) show representative actin stress fiber of the pre-osteoblast MC3T3-E1 cells grown on an uncoated Co-Cr substrate and Ti-deposited Co-Cr after culturing up to 72 h. At 24 h, actin stress fibers were consistently apparent parallel direction following the main cellular axis on Ti-deposited Co-Cr, whereas, actin stresses fiber were weakly developed on Co-Cr. Actin stress fiber was thicker and well organized on Ti-deposited Co-Cr compared to that on bare Co-Cr substrates. The evidence of better actin stress fibers organization on Ti-deposited Co-Cr examined more closely by culturing to 72 h. Actin stress fibers were well defined on Ti-deposited Co-Cr (Figure 4D). Actin stress fiber traversed the entire cross section of the cell. Furthermore these cells exhibited bigger cell shape. Tubulin and actin is the main components of cytoskeleton and cytoskeleton plays critical roles in the control of many aspects of cellular activities, including proliferation, intracellular signaling, cell movement and cell attachment, cytokinesis and endocytosis (Dalby 2005; Pollard et al. 2000). These data suggest that Ti-deposited Co-Cr showed enhancing osteoblast activity significantly.

**Figure 5:**
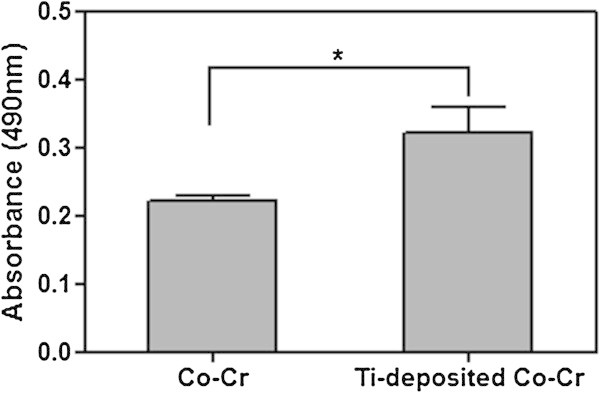
**Proliferation of the osteoblasts on Co-Cr and Ti-deposited Co-Cr 10 days of culturing.** The error bar indicates one standard deviation (n = 3). Statistically significant: ^*^p < 0.05.

Biocompatibility of nanostructured Ti films was further characterized by conducting the cell proliferation analysis. Figure 5 shows the cell proliferation on the uncoated Co-Cr substrate and Ti-deposited Co-Cr. The absorbance values of the cells on the Ti films consistently higher than those on the Co-Cr alloy after all the time periods up to 10 days (p < 0.01) (Figure 5). In particular, the rate of cell proliferation on the Ti-deposited Co-Cr for 10 days culturing was much higher than that on the uncoated Co-Cr by a factor 1.5. The deposition of Ti films increased the absorbance significantly in MTS assay, in which MTS reagent is a pale yellow substance that is reduced to a dark blue formosan product when incubating with viable cells (O’Connor et al. 1990). Therefore, the higher absorbance value was observed on Ti films indicating that the Ti films supported the cell proliferation without cytotoxic effect.

**Figure 6:**
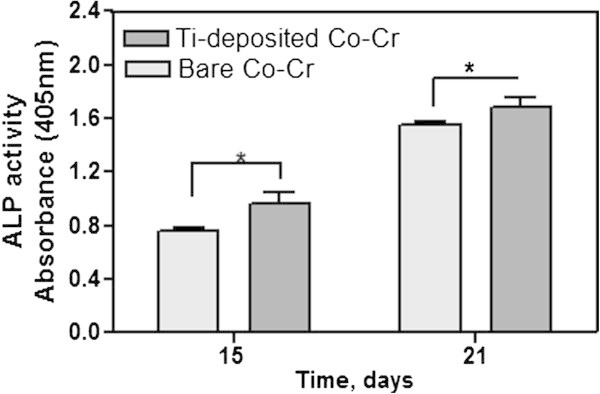
**ALP activity of MC3T3-E1 cells cultured on Co-Cr and Ti-deposited Co-Cr for 15 days and 21 days.** The error bar indicates one standard deviation (n = 3). Statistically significant: ^*^p < 0.05 and ^**^p < 0.01.

The pre-osteoblasts cells (MC3T3-E1) grown in the presence of serum and ascorbic acid differentiate into osteoblasts and produce an extensive collagenous extracellular matrix that can be mineralized by the addition of β-glycerophosphate (Beck et al. 1998). Therefore, the cells were cultured in the medium to induce differentiation. The ALP activity was examined after culturing up to 21 days to determine the effect of the nanostructured Ti films on the cell differentiation. Results of ALP activity on Ti-deposited Co-Cr also showed higher osteoblast activity than that on Co-Cr after all the time periods up to 21 days (p < 0.05) (Figure 6). In particular, the ALP activity level of pre-osteoblasts on the Ti-deposited Co-Cr for 15 days and 21 days of culturing was much higher than that on the uncoated Co-Cr by a factor of 1.4 and 1.2, respectively. The deposition of Ti films increased the ALP activity significantly, which is a cell surface glycoprotein that is involved in mineralization and is the most widely recognized marker of osteoblastic differentiation (Li et al. 2008). This suggests that the Ti films facilitated the differentiation of MC3T3-E1 cells significantly, which was attributed mainly to the application of the highly biocompatible Ti nanostructure on the Co-Cr implants.

## Conclusions

The effect of a nanostructured Ti film on the biocompatibility of a Co-Cr substrate was examined. DC sputtering allowed the successful deposition of a dense and uniform Ti films on Co-Cr. The surface roughness of the Co-Cr was increased remarkably by nanostructured Ti film. Furthermore, the Ti film enhanced the attachment, proliferation and differantiation of osteoblasts remarkably. This suggests the potential use of Ti-deposited Co-Cr as orthopaedic and dental implants.
